# Sourdough Fermentation of Carob Flour and Its Application in Wheat Bread

**DOI:** 10.17113/ftb.58.04.20.6892

**Published:** 2020-12

**Authors:** Dubravka Novotni, Nika Mutak, Ljiljana Nanjara, Saša Drakula, Nikolina Čukelj Mustač, Bojana Voučko, Duška Ćurić

**Affiliations:** 1 University of Zagreb, Faculty of Food Technology and Biotechnology, Pierottijeva 6, 10000 Zagreb, Croatia; 2 University of Applied Sciences ‘Marko Marulić’, Petra Krešimira IV 30, 22300 Knin, Croatia

**Keywords:** antioxidant activity, carob sourdough, dietary fibre, partially baked frozen bread, total phenolics

## Abstract

**Research background:**

Carob is widely cultivated Mediterranean plant, but its flour is scarcely used in bread making. Previous studies investigated the quality of wheat bread with added carob flour showing discrepant results. This study aims to investigate the fermentation performance, antioxidant activity, rheological behaviour and baking application of carob sourdough.

**Experimental approach:**

Carob sourdough was fermented with *Lactobacillus brevis* or *Lactobacillus fermentum* combined with *Saccharomyces cerevisiae* for 24 h at 30 °C. At the end of sourdough fermentation, number of viable lactic acid bacteria and yeast cells, total titratable acidity, pH value, antioxidant activity, phenolics and sugar content were determined. Carob flour (12% flour mass fraction) or sourdough equivalent (22.5% dough mass fraction) was applied in making composite partially baked frozen bread. Dough rheology was monitored using a farinograph. Nutritive value, physical properties and sensory attributes of the rebaked bread samples were evaluated using a hedonic test.

**Results and conclusions:**

By the end of fermentation, carob sourdough reached pH=4.2-4.5 and total acidity 9.9-12.3 mL of 0.1 M NaOH, sugar content on dry mass basis was reduced by 8 g/100 g, while total phenolics and antioxidant activity were increased up to 21%, depending on the starter culture. Addition of carob flour or sourdough to wheat dough resulted in firmer consistency, longer development time, and lower quality number. Regardless, bread with carob flour had significantly improved specific volume. Compared with common wheat bread, carob bread had increased dietary fibre content (46%), total phenolics (140-200%) and antioxidant activity (240-300%), higher shape, larger volume, reduced crumbliness, unchanged firmness and darker crumb colour. Consumer preference and overall acceptability scores of carob sour bread were comparable to those of wheat bread, falling into the category of ’liking very much’.

**Novelty and scientific contribution:**

To our knowledge, this is the first study that proved the feasibility of carob sourdough fermentation using mixed starter cultures, where *L. brevis* together with *S. cerevisiae* was better adapted to the substrate than *L. fermentum*. The carob sourdough could be used as a natural ingredient for improvement of nutritive value and reduction of crumbliness of partially baked frozen bread.

## INTRODUCTION

As a staple food, bread provides complex carbohydrates, proteins, minerals and vitamins in human nutrition, but is poor in different bioactive compounds such as dietary fibre and polyphenols. A current trend is enrichment of bread with natural sources of functional ingredients, such as fibre and polyphenols. The consumption of enriched bread might contribute to the prevention of non-communicable diseases such as obesity, coronary heart-related diseases, type-II diabetes and certain cancers (*1*-*3*). Carob flour, an ingredient typical for the Mediterranean diet, is an example of raw material naturally high in dietary fibre and phenolic compounds. A synergy of carob fibre and polyphenols in prevention of coronary heart diseases and cancer (*2*), as well their potential to lower postprandial blood glucose and insulin was previously demonstrated (*4*). Ortega *et al.* (*1*) showed that the soluble dietary fraction of carob enhances the stability of the phenolic compounds during the duodenal digestion phase. Carob pod is composed of pulp which is high in dietary fibre and polyphenols, and seeds which are rich in proteins and dietary fibre (*2*, *3*, *5*). The seed endosperm has a high content of galactomannans and is used as a source of locust bean gum (E410) for bakery industry, particularly for frozen bread (*6*). Locust bean gum production results in a waste (hulls, germ); thus, the use of wholemeal flour (pulp with seeds) might benefit the production efficiency. This indicates that the use of flour from whole carob pods including both pulp and seeds might be advantageous for bread making.

Nonetheless, carob flour is traditionally used mainly as a cake ingredient, which makes its bread-making performance uncertain. Šoronja-Simović *et al.* (*7*) showed that the addition of 20% carob flour does not favour textural characteristics of wheat bread, but acts as a natural preservative in suppressing microbiological contamination during storage. Salinas *et al.* (*5*) found that bread with increasing mass fractions (from 10 to 30% of carob flour from seed germ and fruit pulp have lower specific volume and higher crumb firmness and chewiness than wheat bread. Çaǧ Lar *et al.* (*8*) established the consumer preference of traditional Turkish soup when carob flour replaced only 3% of wheat flour in tarhana, whereas Herken and Aydin (*9*) claimed successful usage of carob flour up to 15%. In recent years, carob pod extracts have been used in many studies to produce ethanol, citric acid, lactic acid, mannanase, microbial cell protein and other products [REMOVED HYPERLINK FIELD]by fungi, bacteria or algae (*10*). Due to its composition, carob flour might be a suitable substrate for sourdough fermentation, which is still unexploited. Sourdough is known to improve flavour, structure, shelf-life and nutritional value of bread (*11*, *12*). In most industrial processes, sourdough is fermented with selected starter cultures and used as bread improver, while dough leavening is assured by adding baker’s yeast *Saccharomyces cerevisiae*.

This study aims to investigate the possibility of sourdough fermentation of wholemeal carob flour (containing pulp and seeds) with typical heterofermentative sourdough cultures *Lactobacillus brevis* or *L. fermentum*, together with yeast *Saccharomyces cerevisiae*. Sourdough application was tested in making wheat bread. The nutritional, physical and sensorial properties of bread containing carob sourdough (22.5%) or carob flour were compared with control white wheat bread. Bread was partially baked and frozen as is today’s common practice in bakery industry, offering consumers a wide range of freshly baked bakery products throughout the whole day.

## MATERIALS AND METHODS

### Flour

Commercial white wheat flour (Mlin Katić, Brezovica, Slovenia) contained 12% moisture, 12.5% proteins, 33% wet gluten and 0.5% ash. It showed 63.5% of farinograph water absorption and amylograph maximum viscosity 2490 BU according to ICC standards 115/1 (*13*) and 126/1 (*14*), respectively.

Carob flour made of whole raw pods (OPG Božanić, Komiža, Croatia) contained 8.9% moisture, 5.2% proteins, 2.9% ash and 0.6% fat. After determination of sugar and fibre content, starch was calculated by subtracting the content of moisture, proteins, ash, fat, dietary fibre and sugar from 100%. Particle size distribution of carob flour was determined according to Benković *et al.* (*15*) with a laser diffraction system (Malvern Instruments, Worcestershire, UK).

### Carob sourdough fermentation and cell counting

MRS broth (de Man, Rogosa and Sharpe, Biolife, Milano, Italy) was inoculated with the overnight culture of *Lactobacillus fermentum* DSM 20052 or *L. brevis* DSM 20054 (DSMZ GmbH, Braunschweig, Germany) and incubated for 48 h at 37 °C. Malt extract broth (Difco, Saint-Ferreol, France) was inoculated with an overnight culture of *Saccharomyces cerevisiae* and incubated for 48 h at 30 °C. After centrifugation (Rotina, Hettich, Kirchlengern, Germany) at 2000×*g* for 10 min, microbial cells were used as inoculum.

Carob flour (150 g) was mixed with water (450 g, corrected for inoculum volume) and the inoculum of *L. fermentum* or *L. brevis* (approx. 6 log colony forming units (CFU)/g) each combined with yeast *S. cerevisiae* (approx. 4 log CFU/g). Dough yield of 400 was necessary to obtain adequate consistency and sugar concentration. Sourdough was fermented (in duplicate) for 24 h at 30 °C with constant stirring, after which it was stored at 4 °C and used for baking within 2 h or lyophilized for analyses.

Viable lactic acid bacteria (LAB) and yeast cells were enumerated in the inoculum and in sourdough under anaerobic conditions at 30 °C following the ISO 15214:1998 (*16*) and ISO 21527-2:2008 standards (*17*).

### Laboratory bread making

Four bread formulations were tested: control wheat and three types of carob bread (one with carob flour and two with carob sourdough differing in *Lactobacillus* culture). The recipe (expressed as flour mass) for the control bread consisted of wheat flour (100%), farinograph water (63%), compressed yeast (2.5%), salt (1.5%), and sugar (1.9%) according to ICC standard 131 (*18*). For making carob bread, wheat flour was partially replaced with carob flour (12% of total flour, corrected for the moisture content) or equivalent mass fraction of carob sourdough on dough basis (22.5%), while sugar was omitted, and the water addition was corrected for the amount already contained in the sourdough. The recipe for sourdough bread was based on our previous study (*19*), demonstrating that the sourdough fermented with mixed starter cultures has the best effect on improving the quality of partially baked frozen bread by 22.5%. All ingredients were mixed slowly for 2 min and fast for 7 min in a spiral mixer SP12 (Diosna Dierks & Söhne GmbH, Osnabrück, Germany). After resting for 30 min, the dough was divided to 70-gramme pieces and proofed in a fermentation chamber (Wiesheu GmbH, Großbottwar, Germany) at 30 °C and 80% relative humidity for 60 min. In two sets, thirty-six pieces of bread were prebaked in a deck oven preheated at 250 °C, and set at 180 °C for 12 min, with initial steaming (1.9 dm^3^/m^3^). Partially baked bread, previously cooled at ambient conditions to 20 °C, was frozen in a blast freezer (Everlasting, Suzzara, Italy) and packaged in polyethylene bags. After storage at -18 °C for 30 days, and defrosting for 20 min at ambient conditions, the bread was rebaked for 11 min at 220 °C with 50 mL of steam.

### Chemical analyses of carob flour, sourdough and bread

All chemical analyses were done in duplicate. Total titratable acidity (TTA) and pH value of sourdough or bread were determined on 10 g of a sample homogenized with 90 mL of distilled water. TTA is expressed as the volume of 0.1 M sodium hydroxide solution necessary to adjust the pH of 10 g of sample in 90 mL of distilled water to 8.5 (*19*).

Moisture content was determined by two-step drying according to AACC method 44-15.02 (*20*). Total sugars were determined according to the AOAC method 939.03-1939 (*21*). Insoluble dietary fibre, fibre soluble in water but precipitated in 78% aqueous ethanol (SDFP), and fibre soluble in water and not precipitated in 78% aqueous ethanol (SDFS) were determined according to AOAC method 2011.25 (*22*) with integrated total dietary fibre assay kit (Megazyme, Bray, Ireland). SDFS analysis was performed on HPLC system (Shimadzu, Kyoto, Japan) with MetaCarb 67C column (Agilent, Santa Clara, CA, USA).

Total phenolic compounds (TPC) and antioxidant activity were measured spectrophotometrically (with Specord 50 Plus; Analytik Jena, Jena, Germany) after extraction. Sample (1 g) was mixed with 25 mL of acidic methanol/water (*φ*=0.5, pH=2) in a test tube. Tubes were vortexed for 3 min, shaken in a water bath (Stuart®, Cole-Parmer, Staffordshire, UK) for 1 h at room temperature and centrifuged (Rotina, Hettich, Kirchlengern, Germany) at 2500×*g* for 10 min to recover the supernatant. Vortexing, shaking and centrifugation were repeated with another 20 mL of acetone/water (*φ*=0.7) added to the residue. Combined methanolic and acetonic extracts were centrifuged at 3500×*g* for 15 min to obtain the supernatant for the determination of TPC and antioxidant activity. TPC were determined with Folin–Ciocalteu assay according to Durazzo *et al.* (*23*). Diluted extracts (50 µL of carob flour or sourdough extract mixed with 1550 µL of water and 500 µL of bread extract with 1100 µL of water) were mixed with Folin–Ciocalteu reagent (100 µL) and 300 µL of sodium carbonate solution (20%, *m*/*V*). After 2 h of reaction at room temperature, the absorbance was measured at 760 nm against a blank. Gallic acid was used as the standard and results are expressed on dry mass basis as gallic acid equivalents (GAE) per 100 g.

Antioxidant activity assessment was based on the reduction of iron(III)-TPTZ (2,4,6-tripyridyl-striazine) complex to iron(II)-TPTZ at acidic pH by means of ferric reducing antioxidant power (FRAP) as described earlier by Čukelj *et al.* (*24*). Freshly prepared working reagent (1000 µL) was mixed with a sample diluted in *φ*(methanol, acetone)=0.5 (50 µL of carob flour or sourdough extract and 950 µL of diluent, or 200 µL of bread extract and 800 µL of diluent). After 4 min of incubation at 37 °C, the absorbance was measured at 595 nm. A calibration curve was made with known concentrations of Trolox and results are expressed on dry mass basis as Trolox equivalents (TE) per 100 g.

### Determination of physical properties of dough and bread

A farinograph (Brabender GmbH & Co, Duisburg, Germany) was used to determine the water absorption, development time and stability of the dough systems (*13*).

Bread physical properties of five rebaked samples were determined after cooling for 1 h at ambient conditions in minimum five replicates. Volume of weighed sample was measured using the AACC 10-05.01 rapeseed displacement method (*25*). Bread specific volume was calculated as volume to mass ratio. Bread height and diameter were measured by calliper and their ratio presents shape. Crumb firmness was measured according to AACC method 74-09.01 (*26*) with a texture analyser TA.HDplus (Stable Micro Systems, Surrey, UK) using a 25-mm probe. Crumb colour parameters (lightness *L*^∗^, redness *a*^∗^, and yellowness *b*^∗^) were evaluated with a colorimeter (spectrophotometer CH-3500 D; Konica Minolta, Milton Keynes, UK) in the CIELab system (*27*). The breadcrumbs resulting from repeated slicing into 2.5 cm thick slices were collected and weighed (*28*). The crumbliness is expressed in percentage (%) as the mass of the crumbs divided by the mass of the bread.

### Sensory evaluation

Small scale consumer test was run with sixteen non-trained panellists (13 females and 3 males, aged 20–50), the employees of the Faculty of Food Technology and Biotechnology, Zagreb, Croatia, who were familiar with the main bread characteristics. For the 9-point hedonic test the sensory attributes selected were: appearance, smell, taste, texture and overall perception (*29*). A quarter of each sample, including the crust and crumb, was presented to panellists with 3-digit random numbers at the same time. Liking was scored in the range from 1 (dislike extremely) to 9 (like extremely). Moreover, participants ranked bread samples according to the degree of preference from 1 (the most) to 4 (the least). They were also asked if they generally like carob.

### Data analyses

Experimental results were submitted to analysis of variance (ANOVA) to identify statistically significant differences (p≤0.05) between bread samples using Statistica v. 12 (*30*). Sensory data were subjected to ANOVA to test statistically significant differences (p≤0.05) among panellists. Friedman’s ANOVA and Kendall concordance were used to compare sample ranking. Post-hoc Tukey's test indicated significant differences (p≤0.05) between mean values.

## RESULTS AND DISCUSSION

### Carob flour characteristics

The main components of carob flour used in these experiments were sugars (Table 1), of which sucrose was on dry mass basis (45±3) g/100 g. This agrees with Benković *et al.* (*15*), who reported that Croatian carob flour consisting of pulp and seeds contains sucrose ranging from 37 to 38 g/100 g, together with fructose 5-19 g/100 g and glucose 2-21 g/100 g, all on dry mass basis. Carob flour used in this work contained high mass fraction on dry mass basis of dietary fibre (33.2 g/100 g), of which insoluble was 28.1 g/100 g, SDFP 4.5 g/100 g and SDFS 0.6 g/100 g. It was low in starch (4.28 g) and rich in TPC (Table 1), which is in accordance with the previous study (*15*). The fibre content and composition of carob flour widely vary depending on the part of pod used in milling (*3*, *23*), but is comparable to other legume flour types (*11*).

**Table 1:** Total titratable acidity (TTA), total sugars, total phenolic content (TPC), and antioxidant activity (FRAP) of flour, carob sourdough at the end of fermentation, and the resulting bread samples (mean value±standard deviation)

Parameter	Flour		Sourdough		Bread			
Control wheat	Carob flour	*L. brevis* sourdough	*L. fermentum* sourdough	Control bread	Bread with carob flour	Bread with *L. brevis* sourdough	Bread with *L. fermentum* sourdough
pH	-	(5.50±0.10)^d^	(4.19±0.08)^a^	(4.55±0.31)^b^	(5.72±0.01)^A^	(5.50±0.01)^B^	(5.16±0.01)^D^	(5.21±0.01)^C^
TTA (*V*(NaOH)/mL)	-	-	(12.2±0.4)^a^	(9.85±0.07)^b^	(2.7±0.2)^A^	(3.9±0.1)^B^	(5.15±0.07)^D^	(4.6±0.3)^C^
*w*(sugar)/(g/100 g)	(1.11±0.05)^a^	(52.9±2.0)^c^	(45.1±2.1)^b^	(44.4±2.2)^b^	(2.8±0.1)^A^	(5.7±0.3)^C^	(5.2±0.5)^B^	(5.0±0.4)^B^
*w*(TPC as GAE)/(g/100 g)	-	(2.1±0.2)^a^	(2.4±0.1)^b^	(2.5±0.2)^b^	(0.10±0.01)^A^	(0.24±0.01)^B^	(0.27±0.01)^C^	(0.30±0.01)^C^
FRAP *b*(TE)/(mmol/100 g)	-	(2.71±0.04)^a^	(2.92±0.04)^b^	(3.27±0.01)^c^	(0.05±0.01)^A^	(0.17±0.01)^B^	(0.19±0.01)^D^	(0.20±0.01)^C^

*Mean values with different letters in superscript within the same row differ significantly (p<0.05). Lower- and upper-case letters indicate two different data sets

Carob flour showed unimodal asymmetrical particle size distribution with median diameters of 90th percentile d[90]=(431±23) μm, 50th percentile d[50]=(187±7) μm, and 10th percentile d[10]=(38±2) μm. Tsatsaragkou *et al.* (*31*) showed that carob flour particle size affects its dietary fibre and protein content, as well as the volume, porosity, colour and *in vitro* starch digestibility of gluten-free bread. We selected a carob flour fraction with d[50]=175 μm similar to theirs for making high-quality gluten-free bread.

### Sourdough characteristics and chemical properties of bread

To our knowledge this is the first study of the carob sourdough fermentation. The typical heterofermentative LAB strains *Lactobacillus brevis* or *L. fermentum* were used together with yeast *Saccharomyces cerevisiae* to inoculate the sourdough. Sourdough was evaluated by the measurement of microflora CFU, pH and acidity, which are the main criteria for effective fermentation. Generally, a sourdough contains a variable number of LAB and yeasts, ranging from 10^7^ to 10^9^ and 10^5^ to 10^7^ CFU/g, respectively, with a ratio of about 100:1 (*32*). At the end of carob flour fermentation, the count of *L. brevis* was 8.40 log CFU/g, whilst of *L. fermentum* it was 7.76 log CFU/g. *S. cerevisiae* count was independent of the concomitant LAB strain; at the end of fermentations it was (5.8±0.1) log CFU/g on average. Both LAB had a lower cell yield and growth rate in carob than in other legumes (*11*). This may be explained by much higher content of sugars in the carob (but not maltose) and phenolics, but lower of proteins than in other leguminous and cereal flour types. The substrate nutrients and growth factors as well as substrate-derived enzymatic activities are key determinants of the microbial ecology of conventional and gluten-free sourdough (*12*). Phenolic acids inhibit the growth of lactobacilli at concentrations ranging from 0.5 to 4 g/L, depending on the strain (*12*).

Regardless of relatively low LAB and yeast count, carob flour showed satisfactory acidifying capability. At the end of carob fermentation, pH values were substantially lower (Table 1) than the initial pH=5.5. *L. brevis* sourdough had statistically (p<0.05) lower pH and higher TTA than *L. fermentum* sourdough, which is consistent with the cell numbers. The difference between carob flour and sourdough in pH and TTA was reflected in the resulting bread samples; likewise, bread with carob flour was more acidic than the control wheat bread (Table 1). The measured TTA values of carob sourdough are similar to those previously reported for chestnut, millet and rice sourdough, but lower than it is usual for rye, wheat, amaranth, quinoa, or some other legume sourdough (*11*, *33*, *34*). Curiel *et al.* (*11*) after fermentation of different Italian legume flour types with *L. plantarum* C48 and *L. brevis* AM7 strains measured similar pH values (from 4.0 to 4.4), but much higher TTA values, between 20 and 27 mL 0.1 M NaOH, as well as higher cell number of LAB (9.8-10.2 log CFU/g). This just confirms that sourdough acidity and microbial ecology depend on numerous endogenous and exogenous factors (*12*).

During carob fermentation, from starting sugar mass fraction, approx. 8 g/100 g of sugars were depleted (Table 1). Compared with the control wheat bread, the sugar content was on average 84% higher in sour bread samples and 108% higher in the bread containing carob flour. The difference in total sugar content between the starters of sourdough and sour bread was insignificant, although previous studies showed difference in the metabolism of individual sugars. Kim *et al.* (*35*) showed that *L. brevis* simultaneously consumes numerous carbon sources and lacks normal hierarchical control of carbohydrate utilization. Conversely, *L. fermentum* sequentially uses first glucose and fructose, and then ferments sucrose only partially before cessation of its growth (*36*). The major metabolic products formed from glucose and fructose by heterofermentative LAB are lactic and acetic acids, ethanol and mannitol, while *S. cerevisiae* ferments glucose to ethanol and CO_2_ (*16*, *32*). In cereal sourdough, *L. brevis* and *L. fermentum* form a stable communion with *S. cerevisiae* (*16*, *32*). In carob flour fermentation, it was necessary to add yeast since no souring occurred by LAB in its absence (data not shown). This could be due to the yeast’s invertase. Indeed, Turhan *et al.* (*37*) suggested using invertase pretreatment for conversion of sucrose from carob pod extracts into the monosaccharides for lactic acid production by *L. casei*.

Carob flour was rich in TPC (Table 1), which is in agreement with previous study (*15*). Carob polyphenols including phenolic acids (such as gallic and caffeic acids), gallotannins and flavonoids (+)-catechin and (-)-epicatechin (*2*) are able to selectively modify the growth of susceptible microorganisms (*12*). *L. fermentum* strains show a remarkable sensitivity to 4-hydroxybenzoic acids such as gallic acid (*38*) or the phenolic extracts consisting of (+)-catechin and (−)-epicatechin (*39*) and other phenolic compounds (*40*), which affect its growth and fermentative activity. Still, *L. fermentum* is able to decarboxylate caffeic acid into 4-vinylcatechol or reduce it into dihydrocaffeic acid (*41*). Caffeic acid is also present in carob flour but in lesser amount than the gallic acid (*2*). On the other hand, *L. brevis* strains have the ability to decarboxylate gallic acid to pyrogallol (*42*). This could explain the lower growth and acidification rate of *L. fermentum vs*. *L. brevis* in the carob substrate. Compared with the carob flour, TPC significantly increased (p=0.03) after sourdough fermentation with *L. brevis* (21%) and *L. fermentum* (16%). In accordance with our results, Curiel *et al.* (*11*) reported that the sourdough fermentation significantly affects the TPC extractable from different legume flour types. The activity of strain*-*specific esterases, glucosidases, phenolic acid decarboxylases and phenolic acid reductases is responsible for the conversion of phenolic compounds during sourdough fermentation (*12*). Yet, in our work the difference in TPC between sourdough samples with different starters (Table 1) was statistically insignificant (p=0.38).

The bread enriched with carob flour or sourdough had about 3-fold higher content of TPC than the control bread (Table 1). This is in agreement with Turfani *et al.* (*43*) who found a similar increase after adding raw carob flour, although our results for TPC were higher for both control and enriched bread. Moreover, we found that bread samples with carob sourdough had significantly higher TPC than the bread containing carob flour (Table 1). We can assume that not only the amount but also the composition of polyphenols changed with the addition of carob to wheat bread (*24*).

The extractable phenolic compounds are responsible for the antioxidant capacity. FRAP antioxidant activity of carob flour (Table 1) was in the range of previously reported results (*14*). Similar as TPC, antioxidant activity of carob flour significantly (p<0.001) increased after sourdough fermentation with yeast and *L. brevis* (8%) or *L. fermentum* (21%). Curiel *et al.* (*11*) demonstrated a comparable increase in the content of antioxidants after fermentation of different types of legume flour. Generally, the phenolic antioxidants in bakery ingredients are present at relatively low amount (*44*). Processing including proofing and baking may alter the content of phenolic antioxidants in bread to different extents. Still, wheat bread had low antioxidant activity (Table 1), but it was significantly (p<0.001) up to 3-fold higher in bread samples containing carob flour. The higher antioxidant activity of carob sourdough than of carob flour was reflected in the bread samples. Consistent with the increase of TPC, antioxidant activity of the bread with *L. fermentum* sourdough was slightly higher than of that with *L. brevis* sourdough. In agreement with these results, Herken and Aydin (*9*) found that antioxidant capacity and TPC of tarhana increased after adding 10% carob flour.

Carob soluble fibre stabilizes the phenolic compounds during the digestion (*1*). Wheat bread contained 3.5 g/100 g of dietary fibre, whereas all carob bread samples contained (5.1±0.3) g/100 g of which 33% were soluble. The difference in fibre content between carob-containing bread samples was insignificant. Herken and Aydin (*9*) established increased total dietary fibre of tarhana after partial substitution of wheat flour with carob flour. The high mass fraction of polyphenols in carob fibre differentiates it from other dietary fibre sources. Since the average intake of fibre among Western population is generally lower than the recommended, it can be expected that the consumer demand for bread enriched with fibre and phenolic antioxidant contents will continually grow. Thus, the usage of carob flour or sourdough in bread making might have several nutritive advantages due to its high fibre content, acceptable sugar content and antioxidant power. The possibility of carob phytate degradation and improvement of mineral bioavailability with sourdough fermentation still needs to be investigated.

### Dough rheology and physical properties of bread

The influence of a partial replacement of wheat flour with carob flour or sourdough on dough rheology and quality parameters of bread was investigated. Dough rheology during mixing was recorded with a farinograph. Water addition was constant in different recipes and the consistency was monitored (Table 2). Control wheat dough developed the peak consistency after 3.5 min of mixing, which remained almost constant throughout the measuring time. At the beginning of mixing, dough containing carob flour or *L. fermentum* sourdough showed similar consistency as the control; later, their consistency increased for 20-30 BU, and then again softened. Unlike the dough containing carob sourdough, the farinograph quality number of dough containing carob flour can be considered high. Dough containing *L. brevis* sourdough showed the highest consistency, but also a fast softening. The higher consistency indicated a possibility to add more water to make bread with carob flour. The increase of water absorption was low after adding *L. fermentum* sourdough or carob flour (0.5-0.9%), but higher with *L. brevis* sourdough (1.8%). Purhagen *et al.* (*28*) proposed that soluble fibre competes for the water and delays the development of the gluten network. Indeed, dough development time was longer when substituting wheat flour with carob. Still, it was shorter with carob sourdough than with carob flour. Partial acidification of the bread dough strongly influences its mixing behaviour, whereby dough with lower pH values requires a slightly shorter mixing time and is less stable than ordinary dough (*45*). The bandwidth of the farinogram in maximum consistency, as a measure of the dough cohesiveness and elasticity, was the widest for the control dough (70 BU) and remained unchanged during mixing time. Dough containing carob flour or sourdough showed small narrowing of the bandwidth (up to 10 BU, respectively) from the maximum (70 or 60 BU, respectively) towards the end of mixing. Unlike carob, the acidification of wheat sourdough results in a large reduction of elasticity and firmness of the dough (*38*). Our results are in agreement with several studies using carob flour. Šoronja-Simović *et al.* (*7*) and Turfani *et al.* (*43*) showed that the addition of 10-20% commercial carob flour into wheat dough slightly increases the water absorption and prolongs the dough development. Salinas *et al.* (*5*) showed similar farinogram patterns with two peaks of wheat flour blended with 10% carob germ or pulp flour. They also found an increase in water absorption, and higher softening degree of samples with carob flours than the control. Unlike carob pulp flour, addition of 30% carob germ flour leads to the stabilization of dough matrix, indicating some types of carob protein-wheat protein, carob protein-water and lipid-protein interactions (*5*). Evidently, the effect of carob flour or sourdough on dough rheology highly depends on its complex chemical composition.

**Table 2:** Dough rheology and physical properties of the bread with carob flour or sourdough compared with control wheat bread (mean value±standard deviation)

Parameter	Control wheat	Carob flour	*L. brevis* sourdough	*L. fermentum* sourdough
Dough				
Maximum consistency/BU	(500±1)^a^	(528±4)^b^	(558±4)^c^	(520±7)^b^
*t*(dough development)/min	(3.5±0.3)^a^	(12.0±0.4)^d^	(6.0±0.2)^b^	(8.0±0.3)^c^
FQN/mm	>150^a^	(65±2)^b^	(35±2)^c^	(35±3)^c^
Bread				
Baking loss/%	(11.8±0.6)^a^	(11.4±0.6)^a^	(11.6±0.3)^a^	(12.0±0.5)^a^
Specific volume/(cm^3^/g)	(3.1±0.20)^a^	(3.5±0.2)^b^	(3.2±0.1)^ab^	(3.1±0.1)^a^
Shape(expressed as *h*/*d*)	(0.55±0.01)^a^	(0.57±0.02)^ab^	(0.60±0.02)^b^	(0.61±0.02)^b^
Crumb firmness/g	(202.6±9.8)^a^	(197.9±20.7)^a^	(211.4±11.1)^a^	(191.4±15.2)^a^
Crumbliness/%	(1.11±0.14)^a^	(0.05±0.01)^b^	(0.07±0.01)^b^	(0.04±0.01)^b^
Crumb L*	(78.7±0.8)^a^	(58.1±0.7)^b^	(57.0±0.9)^c^	(56.4±0.5)^c^
Crumb a*	(1.1±0.2)^a^	(6.9±0.2)^b^	(7.3±0.3)^c^	(7.5±0.2)^c^
Crumb b*	(21.1±0.4)^a^	(18.8±0.3)^c^	(19.9±0.4)^b^	(20.1±0.4)^b^

Mean values marked with different letters in superscript within the same row significantly differ (p<0.05). FQN=farinograph quality number

The rheological properties of dough are associated with texture, shape and volume of the bread (*44*, *45*). There was no significant difference in baking loss among bread samples (Table 2). The specific volume of bread was improved by 12% when carob flour was added, but it was unchanged upon adding carob sourdough. Contrary, previous studies have shown that fibre-rich material such as carob reduces bread volume and disrupts structure due to the gluten dilution (*43*, *46*). Salinas *et al.* (*5*) concluded that both carob pulp and seed flour (10%) decrease specific volume of wheat bread for about 10%. Šoronja-Simović *et al.* (*7*) showed that wheat bread with 20% added carob flour has 25% lower specific volume than the control bread. The reason why in our experiments carob flour improved the volume could be in fact that bread samples were partially baked and then frozen. During freezing, the volume of partially baked bread contracts. For that reason, hydrocolloids such as locust bean gum from carob seed endosperm are often added in small amounts (0.4-3%) to improve bread volume and shelf-life (*4*, *45*). Our study showed that a volume of partially baked bread can be improved with the addition of wholemeal carob flour. Moreover, bread shape was slightly higher after adding carob sourdough (Table 2), which could be due to dough acidification leading to a more elastic behaviour (*45*). Samples did not significantly differ in crumb firmness (Table 2). Significant correlation between crumb firmness and dough consistency was established (r=0.69). Similarly, Salinas *et al.* (*5*) showed that bread with 10% carob seed flour has unchanged firmness, whereas bread with 10% carob pulp flour had firmer crumb than control bread. Thus, the usage of wholemeal carob flour containing seeds seem advantageous to pulp flour. Softer bread enriched with fibre-rich ingredients can be obtained either by increasing water content or adjusting the baking process (*28*).

Partially baked frozen bread often shows crust defects such as flaking. Crumbliness on cutting significantly (p<0.05) decreased after adding carob flour (26-fold), and even more after adding *L. fermentum* sourdough (30-fold decrease) (Table 2). Contrary, the addition of wheat or rye bran leads to a greater crumbliness (*28*). Hydrocolloids such as locust bean gum from carob seeds can increase crust firmness of partially baked bread and its viscoelastic properties (*46*).

As expected, bread samples with carob flour were visibly darker with significantly (p<0.05) lower L* and yellow pigment b*, but higher redness value a* of the crumb. Bread with carob sourdough had higher intensities of a* and b* than the bread containing carob flour. This could be related to the higher content of phenolic compounds in the sourdough bread. Consistent with our results, lower L* values have been reported after using carob flour for partial substitution of rice in gluten-free bread (*31*) or wheat flour in tarhana (*8*, *9*). In their studies, other colour parameters varied differently, probably because their formulations contained different colourful ingredients. Herken and Aydin (*9*) reported increased a* and b*, whereas Çağ Lar *et al.* (*8*) found that a* and b* values of tarhana decreased with carob flour substitution. Based on the results of our study, we can conclude that carob flour containing seeds, or its sourdough, could be used as a natural ingredient for enrichment of bread with fibre and phenolic compounds without decreasing its technological quality.

### Consumer acceptability of bread

**Fig. 1:**
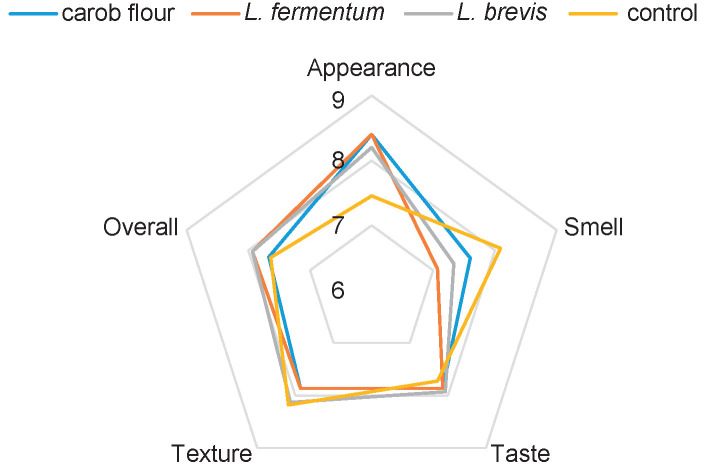
Consumers acceptability of bread with carob flour or sourdough fermented with *Lactobacillus fermentum* or *L. brevis* compared with the control white bread

The consumer acceptability of bread with carob flour or sourdough was investigated. There was no statistical difference among panellists’ acceptability (p=0.40). The only significant difference (p=0.02) between sensory characteristics of bread samples containing carob and the control bread was in their appearance (Fig. 1), probably due to the colour difference. The smell of carob bread is highly specific, therefore most of the panellists liked better the smell of common wheat bread, but the difference was insignificant. For some consumers the taste of bread containing carob flour (but not sourdough) was too sweet. Aguilar *et al.* (*33*) reported reduced sweetness of gluten-free bread containing chestnut sourdough compared with their unfermented controls, which negatively affected consumers preference. The discrepancy between results could be due to the differences in bread type and lower amount of sugars (19%) in their chestnut flour than in carob flour used in this work. The liking of texture correlated with instrumentally measured crumb firmness (r=0.79). Although the overall liking was not significantly different among bread samples, carob sourdough bread was liked the most. Our results agree with several studies. Aguilar *et al.* (*33*) reported that consumers did not perceive any differences in crumb hardness, aroma and taste of the bread made with or without chestnut sourdough. Herken and Aydin (*9*) reported unchanged scores of the overall acceptability of soup made with tarhana supplemented with up to 15% carob flour compared with the control. Çağ Lar *et al.* (*8*) established the unchanged consumers liking of tarhana soup in terms of colour, taste and odour when 3% of wheat flour was replaced with carob flour, but not at 5 and 8% replacement.

The *L. fermentum* sourdough bread was most preferred with the highest number of times having the sum of ranks 37, but its average rank (2.34±1.08) and the sum of ranks were similar to those for *L. brevis* sourdough bread (2.28±1.00) (*36*) and bread with carob flour (2.38±1.03) (*38*). The control bread was the least preferred, ranking on average (3.00±1.29) with the sum of ranks 47. Still, the Friedman’s ANOVA showed no significant differences in preference among the bread samples (p=0.35), and the Kendall’s coefficient of concordance (0.07) indicated the lack of agreement in the ranking of the samples among panellists. This is probably related to the fact that a half of the panellists liked carob and the other half did not.

## CONCLUSIONS

In this study we investigated the possibility of using carob flour or its sourdough for enrichment of wheat bread with dietary fibre and phenolic antioxidants. Sourdough fermentation of carob flour is feasible with mixed starters of lactobacilli and yeast *Saccharomyces cerevisiae*. *Lactobacillus brevis* associated with *S. cerevisiae* is better adapted to carob substrate than *L. fermentum,* which seems to be sensitive to the high amount of phenolic compounds. Sourdough fermentation of carob flour can be recommended for further enhancement of its phenolic content and antioxidant activity, the reduction of sugar content, as well for shortening the dough mixing time. Despite some differences in dough rheology, the choice between starter cultures *L. brevis* or *L. fermentum* does not make major differences in bread quality. Carob flour or sourdough can be used as a natural improver of partially baked and then frozen bread to reduce crumbliness. Volume, crumb texture and consumers acceptability of carob breads is comparable to common white wheat bread. Because of its nutritional benefits, consumer liking, technological suitability and acceptable pricing, fermented carob flour is a promising ingredient for health-promoting bakery foods. Future studies should investigate the influence of different parameters of carob sourdough fermentation and its application in gluten-free bakery products.
